# Zygote cryobanking applied to CRISPR/Cas9 microinjection in mice

**DOI:** 10.1371/journal.pone.0306617

**Published:** 2024-07-09

**Authors:** Geraldine Schlapp, María Noel Meikle, Jorge Luis Pórfido, Alejo Menchaca, Martina Crispo

**Affiliations:** 1 Laboratory Animal Biotechnology Unit, Institut Pasteur de Montevideo, Montevideo, Uruguay; 2 Plataforma de Salud Animal, Instituto Nacional de Investigación Agropecuaria (INIA), Montevideo, Uruguay; 3 Fundacion IRAUy, Instituto de Reproducción Animal de Uruguay, Montevideo, Uruguay; Eidgenossische Technische Hochschule Zurich, SWITZERLAND

## Abstract

Microinjection of CRISPR/Cas9 requires the availability of zygotes that implies animal breeding, superovulation schemes, and embryo collection. Vitrification of zygotes may allow having ready-to-use embryos and to temporally dissociate the workload of embryo production from microinjection. In this study, fresh (F group) or vitrified (V group) zygotes were microinjected with CRISPR/Cas9 system to test the hypothesis that vitrified zygotes could be a suitable source of embryos for microinjection. In Experiment 1 (*in vitro* evaluation), B6D2F1/J zygotes were microinjected and cultured until blastocyst stage. Embryo survival and cleavage rates after microinjection were similar between groups (~50% and ~80% respectively; P = NS). Development rate was significantly higher for F than V group (55.0% *vs*. 32.6%, respectively; P<0.05). Mutation rate did not show statistical differences among groups (P = NS). In Experiment 2 (*in vivo* evaluation), C57BL/6J zygotes were microinjected and transferred to recipient females. Embryo survival was significantly lower in fresh than in vitrified zygotes (49.2% *vs*. 62.7%, respectively; P<0.05). Cleavage rate did not show statistical differences (~70%; P = NS). Pregnancy rate (70.0% *vs*. 58.3%) and birth rate (11.9% *vs*. 11.2%) were not different between groups (F *vs*. V group; P = NS). Offspring mutation rate was higher for F than V group, in both heterodimer analysis (73.7% *vs*. 33.3%, respectively; P = 0.015) and Sanger sequencing (89.5% *vs*. 41.7%, respectively; P = 0.006). In conclusion, vitrified-warmed zygotes present a viable alternative source for CRISPR/Cas9 microinjection when the production of fresh embryos is impeded by limited technical support. The possibility of zygote cryobanking to perform microinjection sessions on demand seems to be a suitable alternative to avoid the breeding and maintenance of animals all over the year, enhancing the implementation of CRISPR technology.

## Introduction

CRISPR/Cas9 system is an efficient, versatile and affordable tool that has emerged as the method of choice to generate valuable genetically engineered animals among the different species and technological platforms worldwide. This technology has been extraordinarily successful in the mouse, leading to a wide spectrum of precise genetically engineered models [1]. Not only by using the CRISPR/Cas9 system, it is possible to delete or insert a given sequence, but also using the CRISPR-associated catalytically inactive dCas9 fused to effector protein domains with different regulatory functions, enables transcriptional repression or activation [2]. Moreover, dCas9 and later, a nicked Cas9 (nCas9) which introduces a nick in one of the DNA strands, have been used to perform base and prime editing, which allows introducing single nucleotide substitutions [3]. The delivery of the CRISPR/Cas9 system into the zygotes is usually performed by pronuclear or cytoplasmic embryo microinjection, but other methods have proven to be successful, for instance electroporation [4], and viral delivery methods such as adeno-associated (AAV) viral vectors [5]. Zygote micromanipulation requires a great number of zygotes ready-to-inject for a particular day or days of the week, which is provided by the maintenance of a colony of donor females with a great number of animals that differ in age and physiological status. Each CRISPR project needs to be associated to zygote donor rearing and preparation, superovulation schemes for *in vivo* or *in vitro* zygote production and embryo collection, which are time-consuming, laborious and imply a tight schedule. Embryo production also implies a continuous availability of stud males all over the year, which could be purchased or generated in-house at the facility. Although it is never said, the procedures related with zygote supply represent an operational limitation that if improved, could greatly simplify the implementation of CRISPR projects.

Cryopreservation is a standard procedure used in mouse facilities to backup genetic lines and to store off-shelf strains. Vitrification is often preferred to slow freezing since it is an easier, faster, and cheaper technique [6, 7]. This technology is a very valuable tool to be considered in the generation of genetically engineered animal models, not only to maintain a cryobank of the developed models, but also to store oocytes and zygotes for further *in vitro* fertilization (IVF) or microinjection. Vitrification of zygotes may allow having ready-to-use embryos for microinjection and to temporally dissociate the workload of embryo production from microinjection. The use of *in vitro* fertilized or mated vitrified-warmed oocytes for CRISPR/Cas9 microinjection [8] or electroporation [9] have been previously reported, showing efficient production of gene knock out and knock in mice -by amino acid substitution-, which incorporates assisted reproduction techniques to the generation of genetically modified mice. Minimum volume vitrification methods have been improved in recent years [10]. In our laboratory, we have successfully implemented the use of minimum volume vitrification systems in mammals’ oocytes, early-stage embryos (e.g., eight-cell embryos) and late-stage embryos (e.g., blastocysts) [11–13]. In mice, the Spatula MVD (Spatula Montevideo) method is used as a routine with some modifications to the original device [14] acquiring greater embryo holding capacity among other refinements [12]. A major advantage of the spatula, it could be home-made in a few minutes in contrast to other vitrification devices that are only commercially available. By using the Spatula MVD method in eight-cell embryos, higher survival rates were reported comparing to slow-freezing method, and similar pregnancy and birth rates were obtained compared to fresh embryos [12]. We propose that zygote vitrification by minimum volume spatula method soon after fertilization can be a useful tool for constantly providing putative zygotes for microinjection.

The objective of this study was to compare the outcomes in the production of CRISPR/Cas9 gene edited mouse models using fresh *vs*. vitrified zygotes prior to microinjection, aiming to propose the cryobanking as an alternative to ensure the availability of zygotes on demand for routine microinjection.

## Materials & methods

### Animals and housing

Mice were bred and housed at the specific pathogen-free (SPF) animal facility of the Laboratory Animal Biotechnology Unit of Institut Pasteur de Montevideo, Uruguay, in individually ventilated cages (IVC-Tecniplast, Milan, Italy) containing chip bedding (Toplit 6, SAFE, Augy, France). They were provided with free access to autoclaved food (5K67, LabDiet, MO, US) and autoclaved filtered water. Environmental conditions were 20±1°C, 30–70% relative humidity and 14/10 light/dark cycle. Mice were provided with nesting and enrichment material: paper towels, cardboard and polycarbonate red houses in an alternate manner. Animals were housed and handled according to national law 18.611 and international animal care guidelines [15]. Experimental protocol (permit number #007–18) was opportunely approved by the Institut Pasteur de Montevideo Animal Care and Use Committee (written consent was given). Embryo transfer surgery was performed under ketamine-xylazine anesthesia, and analgesia was administered prior to the surgery to minimize pain.

### Experimental design

**Fig 1:**
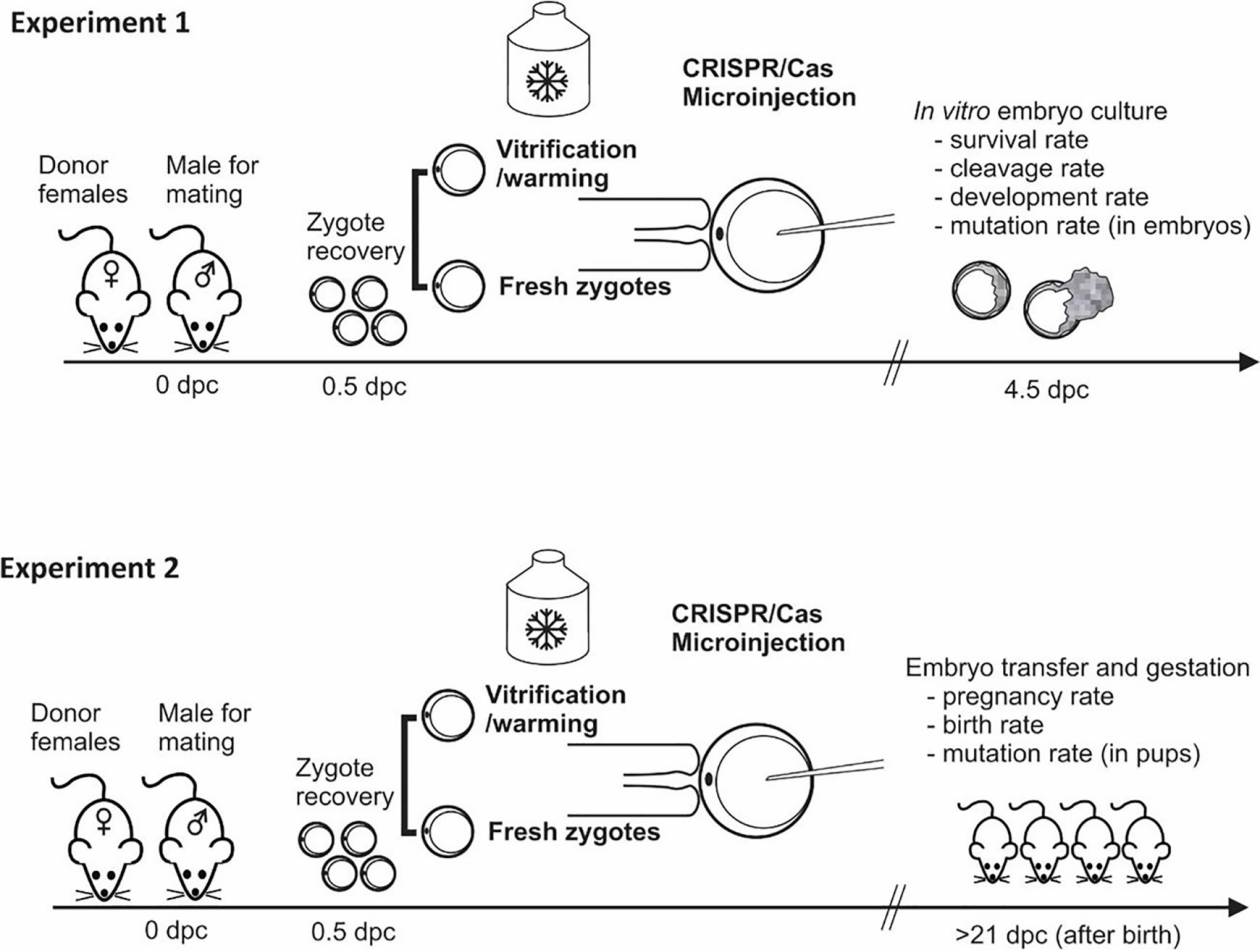
Schematic representation of the experimental design to evaluate the effectiveness of zygote cryopreservation compared with fresh zygotes to be used for CRISPR/Cas9 microinjection.

Experiment 1 (*in vitro* evaluation) and Experiment 2 (*in vivo* evaluation) were performed with the aim to evaluate the effect of zygote vitrification prior to CRISPR/Cas9 microinjection on several variables (Fig 1).

Zygotes were collected from female donors at 0.5 dpc to be subjected to vitrification/warming or to be maintained as fresh zygotes. After that, CRISPR/Cas9 microinjection, using Cas9 ribonucleoprotein (RNP), was performed in both experimental groups. In Experiment 1, embryo development was evaluated under *in vitro* conditions, while in Experiment 2, pregnancy and birth rates were evaluated after embryo transfer in pseudopregnant recipient females. Mutation rate was determined in both experiments, first in embryos and then in pups (Experiment 1 and Experiment 2, respectively).

For both experiments, zygotes were collected at 0.5 days post coitum (dpc) and were immediately microinjected with CRISPR/Cas9 (F group) or subjected to vitrification/warming before microinjection (V group). In Experiment 1, soon after collection, fresh zygotes (n = 313) were microinjected while the other zygotes were vitrified, then warmed and microinjected 4 days later (n = 275) and incubated until blastocyst stage. CRISPR/Cas9 microinjection was performed identically in both experimental groups as described below. In Experiment 2, fresh zygotes (n = 563) were microinjected at 0.5 dpc and transferred to pseudopregnant females at two-cell stage while the other zygotes (n = 558) were vitrified at 0.5 dpc, warmed and microinjected 4 days after cryopreservation, and finally transferred to pseudopregnant females at two-cell stage. Embryo recovery rate (recovered/vitrified embryos), embryo survival rate (live/recovered or /microinjected embryos), cleavage rate (two-cell/live embryos), embryo development (blastocysts/live embryos; blastocysts/two-cell embryos), pregnancy rate (pregnant/transferred females), birth rate (live pups/transferred embryos), and mutation rate (positive/analyzed embryos or pups) were determined according to each experimental design. The experiments were carried out in four different CRISPR projects that were ongoing at the facility. Each microinjection session was managed as a replicate. Experiment 1 was performed on a total of 588 zygotes that were microinjected in four sessions belonging to one CRISPR project (#1). Experiment 2 was carried out in six microinjection sessions belonging to three different projects. In this case, a total of 1121 zygotes were microinjected (project #2, 180 zygotes in one replicate; project #3, 772 zygotes in four replicates; project #4, 169 zygotes in one replicate). The efficiency of CRISPR/Cas9 system was determined in terms of generation of mutant alleles (indel mutations) obtained as a result of NHEJ repair mechanism induced by Cas9 cleavage. Analysis was performed by heterodimer detection in PAGE, in both blastocysts (Experiment 1) and pups (Experiment 2) samples, and then, by Sanger sequencing of samples from live pups. Unless otherwise indicated, chemicals were purchased from Sigma Chemical Company (St. Louis, MO, US).

### Zygote collection

Embryo manipulation at different stages was performed using commercial media, M2 medium (Sigma-Aldrich #M7167) for bench manipulation and M16 (Sigma-Aldrich #M7292) for incubation in 5% CO_2_ in air at 37°C [16]. Zygotes were produced in 3–4 week-old and 8–12 week-old B6D2F1/J hybrid female (C57BL/6J females Jax stock # 000664 x DBA/2J males Jax stock # 000671) (n = 28) for Experiment 1, and in C57BL/6J females (n = 45) of both ages in Experiment 2. The donors received a standard ovarian superstimulatory treatment [17] that consisted of a single dose of 5 IU of equine chorionic gonadotrophin (eCG; Novormon®, Zoetis, Buenos Aires, Argentina) and 5 IU of human chorionic gonadotrophin (hCG; Chorulon®, Intervet International B.V., Boxmeer, Netherlands) 46 h later, both administered via intraperitoneal (ip) injection. Immediately, females were mated with adult C57BL/6J male mice in 1:1 ratio. They were euthanized by cervical dislocation 22 h after mating, the oviducts were excised, and the zygotes were collected from the swollen ampulla in M2 medium containing 0.3 mg/mL hyaluronidase to remove cumulus cells. All ova/zygotes were maintained in M16 medium microdrops under embryo tested mineral oil, in the incubator until microinjection/vitrification.

### Zygote vitrification/warming

After collection, zygotes were evaluated morphologically under stereomicroscope and degenerated zygotes were discarded. Zygotes were subjected to cryopreservation using the home-made Spatula MVD method described previously [12]. For this purpose, zygotes were placed in a pre-vitrification solution containing 10% dimethyl sulfoxide (Me_2_SO), 10% ethylene glycol, and 80% M2 medium, for 30 s and immediately transferred to a vitrification solution (15% Me_2_SO, 15% ethylene glycol, 10% M2 and 60% Ficoll) for another 30 s. Using a glass pipette, zygotes were loaded on the spatula, immediately plunged in LN_2_ and 5 s later inserted into a 0.5 mL straw cap under LN_2_. Spatulas were stored in a LN_2_ dewar until microinjection.

Warming was performed by dipping the spatulas in a 500 µL M2 drop containing 0.5 M sucrose, where the zygotes fall from the spatula. They were immediately washed in 0.5 M and 0.25 M sucrose drops for 2 min each and rinsed in M2 several times. After warming, zygotes were maintained in M16 drops at 37°C and 5% CO_2_ until microinjection.

### CRISPR/Cas9 microinjection

Pronuclear (PN) microinjection of CRISPR/Cas9 RNP was performed using an automatic injector (FemtoJet® 4i, Eppendorf, Hamburg, Germany), an inverted microscope (TE 2000, Nikon, NY, US) and mechanical micromanipulators (Transferman® 4r, Eppendorf, Hamburg, Germany). The workstation is placed over an antivibration table. Embryo holding pipettes were commercially acquired (35° bent angle, opening diameter of 20–25 μm; Biomedical Instruments, Zöllnitz, Germany) and injection pipettes home-pulled (O.D. 1.0 mm, I.D. 0.78 mm, 10 cm length; Sutter Instrument, Novato, CA, US). CRISPR mixes were prepared according to each project, containing: 1.0–2.5 ng/μL sgRNA (Synthego, Redwood city, CA, US), and 2.5–5.0 ng/μL Cas9 protein (QB3 MacroLab, Berkeley, CA, US) diluted in TE buffer (1 mM Tris, pH 8.0, 0.1 mM EDTA; Synthego). sgRNAs were designed employing CRISPOR software (http://crispor.tefor.net/) and chosen according to their relative position to the desired editing site, expected activity and specificity (sgRNA sequences and target genes are detailed in S1 Table). The sgRNAs were validated *in vitro* by treatment of specific PCR products with sgRNA/Cas9 RNP complexes. In each microinjection session, 100–150 zygotes were loaded in groups of 30 in glass depression slides containing M2 medium under mineral oil. The compensation pressure was set at 40 hPa, and the injection pressure initially set at 1000 hPa, which could change depending on the flow of the microinjection mix. During injection, a visible but slight swelling of the PN membrane was observed. After each microinjection series, embryos were transferred to a M2 drop to evaluate viability on a stereomicroscope. Surviving embryos were maintained in M16 drops at 37°C and 5% CO_2_ until 4.5 dpc when embryo development was assessed under *in vitro* conditions (Experiment 1), or until 1.5 dpc (two cell-stage) in the case they were transferred the day after microinjection (Experiment 2). Surviving zygotes were defined according to standard criteria [18].

### Embryo transfer

In Experiment 2, a total of 22 B6D2F1 recipient females were transferred with an average of 20 two-cell embryos each the day after microinjection. Ten females were transferred with fresh microinjected zygotes and 12 ones with microinjected vitrified-warmed zygotes. In order to induce pseudopregnancy, B6D2F1 females were mated with in-house vasectomized adult males with proven sterility and checked for copulatory plugs the day of embryo transfer (0.5 dpc). Recipient females were anesthetized with a ketamine-xylazine mixture (ketamine 110 mg/kg, Pharmaservice, Ripoll Vet, Montevideo, Uruguay; xylazine 13 mg/kg, Seton 2%; Calier, Montevideo, Uruguay) via ip injection. Soon after anesthesia, each female received one dose of tolfenamic acid subcutaneously (1.0 mg/kg, Tolfedine®, Vetoquinol, Lure, France) to provide surgical analgesia [19]. Females were placed over a warm pad to avoid hypothermia, both during surgery and recovery. In average, a total of twenty embryos were transferred to each recipient female through the infundibulum into both oviducts, using a pulled glass pipette and M2 medium [20]. Animals were monitored until full recovery from anesthesia and inspected the day after the surgery. Pregnancy diagnosis was determined by visual inspection by an experienced animal caretaker two weeks after embryo transfer and litter size was recorded on day 21 after birth.

### Genotyping of blastocyst and pups

In Experiment 1, 29 and 30 blastocysts that developed from zygotes of Fresh and Vitrified groups, respectively, were individually subjected to PCR. DNA extraction was performed using ARCTURUS™ PicoPure™ kit (Applied Biosystems–Life Technologies, Carlsbad, CA, US) following manufacturer instructions. In Experiment 2, tail biopsies of all live pups from both experimental groups were taken at weaning and were digested overnight at 55°C using 0.5 mg/mL proteinase K (Life Technologies, Austin, TX, US) in lysis buffer (10 mM Tris pH 8; 100 mM NaCl; 10 mM EDTA pH 8; and 0.5% SDS). On the following day, a 5 M NaCl solution was added to the digestion mix, ice-incubated for 10 min and then centrifuged at 9500 g for 10 min. Supernatant containing DNA was precipitated in cold 95% ethanol. DNA was pelleted and washed with cold 70% ethanol. Pellet was completely dried at 55°C and then resuspended in ddH_2_0. Mutation rate was determined by indel detection in both blastocysts and pups through polyacrylamide gel electrophoresis (PAGE) analysis, and then by Sanger sequencing only in pups. For PAGE analysis, PCR was performed in a Thermal cycler 2720 (Applied Biosystems), using Q5® High-Fidelity DNA Polymerase (New England BioLabs, Ipswich, MA, US). PCR products were checked in GreenSafe Premium (NZYTech, Lisboa, Portugal) stained 1% agarose gel and then subjected to PAGE in 8% polyacrylamide gels for detection of heterodimers [21]. For Sanger sequencing, the pups’ DNA samples were subjected to PCR (using the same Polymerase and cycler) and its products were checked for a single band in a 1% agarose gel electrophoresis. These samples were purified and sequenced by Macrogen, Inc. using custom primers (PCR and sequencing primer sequences are detailed in S2 Table). Sequences were analyzed both manually and with Synthego ICE Analysis Tool (https://ice.synthego.com/#/).

### Statistical analysis

Embryo survival, cleavage, development, pregnancy, birth and mutation rates were compared among groups by logistic regression in generalized linear mixed models (GLMM) using InfoStat 40 software [22]. Experiment 1 was carried out in four microinjection sessions corresponding to one CRISPR project, while Experiment 2 was done in six sessions belonging to three different projects. Each microinjection session was managed as one replicate. For each experiment, the model included the treatment as a fixed variable, while the zygote, replicate and project were included as random variables. To determine the minimum sample size before the experiment the statistical power level was defined at a minimum of 0.80 for each variable, and the statistical significance was defined as P<0.05.

## Results

### *Experiment 1*: Embryo development of zygotes subjected to vitrification/warming prior to CRISPR/Cas9 microinjection

In this experiment, 760 embryos were collected from 28 B6D2F1/J donor females, which were randomly and equally allocated to the experimental groups, including unfertilized oocytes that were excluded at the microinjection stage. For those zygotes subjected to vitrification/warming, recovery rate (recovered/vitrified embryos) from the spatula device was 97.1% (369/380) and embryo survival rate (live/recovered embryos) was 90.8% (335/369). Microinjection was performed in 313 fresh zygotes and 275 vitrified/warmed zygotes. The outcomes after microinjection are summarized in Table 1.

**Table 1:** Experiment 1. Embryo survival, cleavage, development and mutation rate of CRISPR/Cas9 microinjected fresh or vitrified/warmed B6D2F1/J hybrid zygotes.

Group	No. of microinjected zygotes	Embryo survival (live/ microinjected embryos)	Cleavage rate (2-cell/ live embryos)	Development rate (blastocysts/ live embryos)*	Development rate (blastocysts/ two-cell embryos)*	Mutation rate (positive/ analysed embryos)**
Fresh zygotes	313	48.2%	78.8%	55.0%	69.7%	58.6%
		(151/313)	(119/151)	(83/151)	(83/119)	(17/29)
Vitrified zygotes	275	49.1%	80.0%	32.6%	40.7%	36.7%
		(135/275)	(108/135)	(44/135)	(44/108)	(11/30)
*P-*value		0.84	0.80	<0.001	<0.001	0.09

Results are shown as percentages. Number of embryos are shown in parenthesis. * 4.5 days post coitum; only expanded and hatched blastocysts are considered. ** Positive embryos showing DNA heterodimers in polyacrylamide gels.

**Fig 2:**
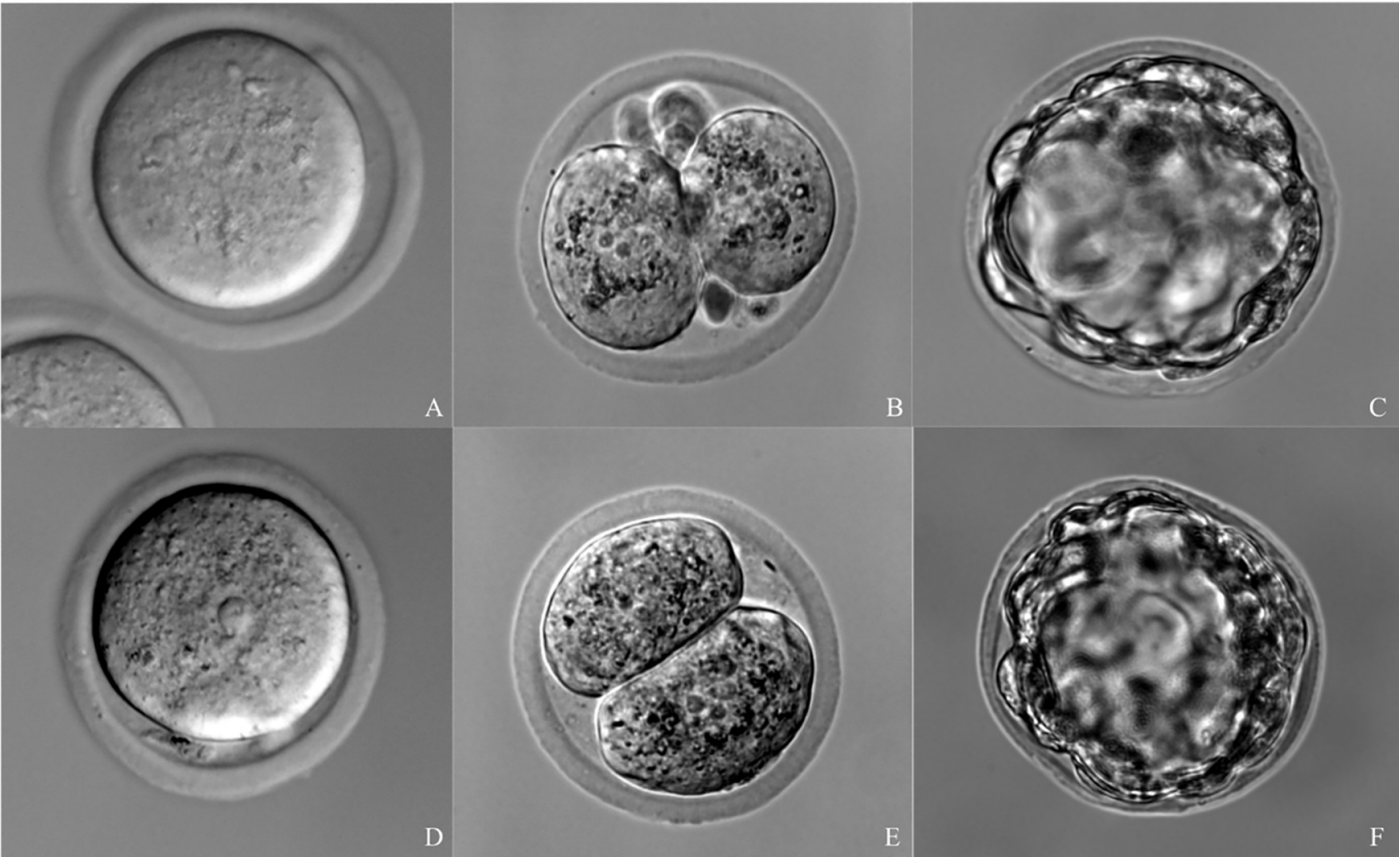
Representative DIC images of *in vitro* developed embryos (Experiment 1). Microinjection of fresh zygotes (A) or after vitrification-warming (D) resulted in two-cell embryos and expanded blastocysts upon incubation (B and C injected as fresh; E and F injected after vitrification-warming).

Embryo survival rate after microinjection and cleavage rate were similar in fresh and vitrified embryos (P = NS), while blastocyst development rate was significantly higher in fresh zygotes (P <0.05). Differential interference contrast (DIC) images of both fresh and vitrified microinjected embryos were obtained at zygote, two-cell and expanded blastocyst stages (Fig 2) using an Olympus IX81 microscope at 40x and a Hamamatsu ORCA-ER digital camera, 12 bits, with a resolution of 1344 (H) × 1024 (V) pixels.

**Fig 3:**
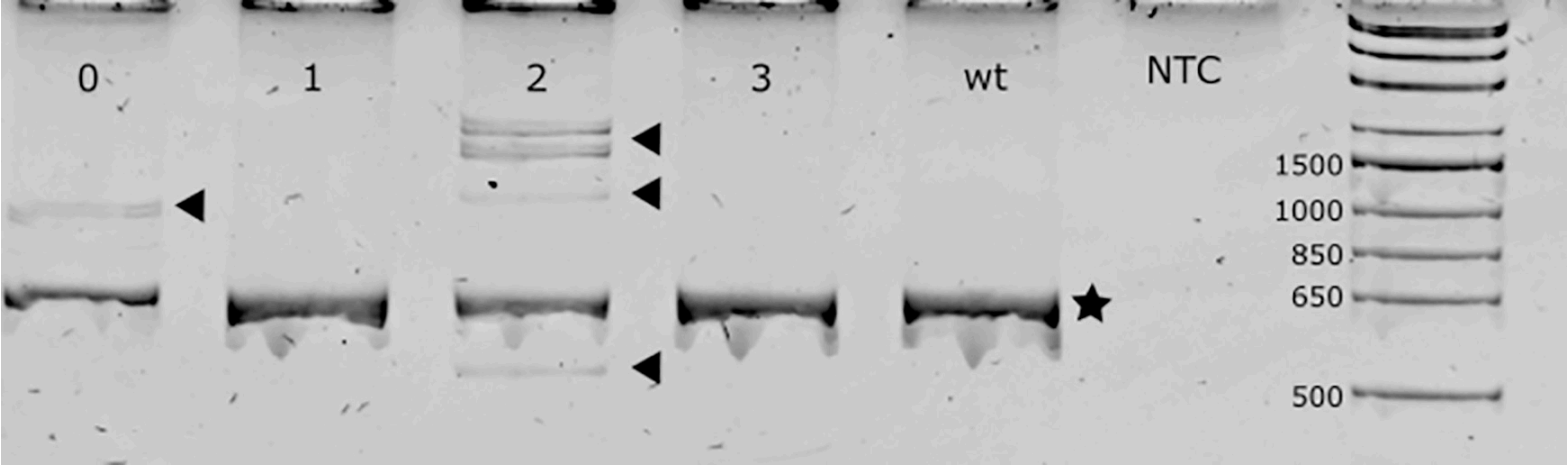
Representative PAGE image. DNA samples were obtained from blastocysts that were microinjected in zygote stage. Samples 0 and 2 were positive for indels; samples 1 and 3 were negative for indels. WT = wild-type blastocyst; NTC = PCR negative control. Arrows indicate heterodimers. Asterisk indicate the wild-type allele. 1 kb plus DNA ladder (Thermofisher) was used.

Mutant rate (heterodimer formation) was detected in 58.6% and 36.7% of fresh and vitrified microinjected embryos, respectively, not showing statistical significance (P = NS). A representative PAGE image is shown in Fig 3.

### *Experiment 2*: Pregnancy and birth rates of zygotes subjected to vitrification/warming prior to CRISPR/Cas9 microinjection

In this experiment, 1600 embryos were collected from 45 C57BL/6J donor females which were randomly and equally allocated to the experimental groups, including unfertilized oocytes that were excluded at the microinjection stage. For those zygotes subjected to vitrification/warming, recovery rate from the spatula device was 84.0% (672/800) and embryo survival rate was 92.3% (620/672). Microinjection was performed in 563 fresh zygotes and 558 vitrified/warmed zygotes. Overall results are summarized in Table 2.

**Table 2:** Experiment 2. Embryo survival, cleavage, pregnancy, birth and mutation rates of CRISPR/Cas9 microinjected fresh or vitrified/warmed C57BL/6J zygotes.

Group	No. of micro- injected zygotes	Embryo survival (live/micro- injected)	Cleavage rate (2-cell/live embryos)	Pregnancy rate (pregnant/ transferred females)	Birth rate (live pups*/ transferred embryos)	Mutation rate^1^ (positive/ live pups**)	Mutation rate^2^ (positive/ live pups**)
Fresh zygotes	563	49.2%	71.7%	70.0%	11.9%	73.7%	89.5%
		(277/563)	(197/277)	(7/10)	(21/176)	(14/19)	(17/19)
Vitrified zygotes	558	62.7%	67.7%	58.3%	11.2%	33.3%	41.7%
		(350/558)	(237/350)	(7/12)	(24/214)	(8/24)	(10/24)
*P-*value		<0.001	0.23	0.58	0.83	0.015	0.006

Results are shown as percentages. Number of embryos or pups are shown in parenthesis. * live pups 7 days after birth.

** live pups after 21 days after birth. Mutation rate^1^ refers to detection of DNA heterodimers in PAGE; mutation rate^2^ refers to detection of indels by Sanger sequencing.

Cleavage rate, pregnancy and birth rates showed no significant differences between both groups (P = NS), while embryo survival was significantly higher in vitrified zygotes than in fresh ones (*P*<0.05). Mutation rate, evaluated in terms of indel formation, was higher in fresh zygotes than in vitrified ones (*P*<0.05), a result that was found firstly by heterodimer detection and then confirmed by Sanger sequencing. Sequencing showed that two more pups in V group and three more pups in F group were also positive, showing 1bp insertions or deletions. A representative sequencing analysis of three samples is shown in S1 Fig. Results of Experiment 2 were also evaluated separately by project (S3 Table). Although there were no significant differences in some of the comparisons, the results of the projects evaluated separately agreed with the results managed jointly.

## Discussion

Overall results showed that cryopreserved zygotes by minimum volume spatula vitrification are suitable for CRISPR/Cas9 microinjection. Vitrified-warmed zygotes survived to microinjection, developed into hatched blastocysts or produced live pups, and finally showed mutations induced by CRISPR/Cas9 system, although the mutation rate was significantly lower in vitrified zygotes than in fresh ones.

Vitrification of presumptive zygotes soon after fertilization (i.e., one-cell embryos) did not affect neither zygote survival to CRISPR/Cas9 microinjection nor embryo cleavage rate after microinjection, which in this study was observed in two different genetic backgrounds (B6D2F1/J and C57BL/6J). Surprisingly, vitrified zygotes showed significantly higher embryo survival than fresh ones, a finding that needs further investigation. Although cryopreservation of oocytes soon after *in vitro* fertilization prior to microinjection with CRISPR/Cas9 system was previously proposed [8], to our knowledge the current results comparing fresh and vitrified zygotes using a vitrification spatula had not been reported in any species. This finding opens new insights to simplify the CRISPR pipeline with the possibility of zygote cryobanking to avoid the need for a facility permanently providing fresh zygotes for microinjection.

Blastocyst development under *in vitro* conditions (expanded/hatched blastocysts over live microinjected embryos) was significantly lower in vitrified embryos than in fresh ones, while no differences were found in birth rate (live pups over transferred two-cell embryos). The lower embryo development rate induced by vitrification was already observed using the same vitrification system in non-microinjected one-cell embryos [14] and eight-cell embryos [12]. Although vitrification has been successfully applied to a variety of mammalian embryos and oocytes, it may cause different abnormalities, for instance blastomere damage and abnormal spindles [23], mitochondrial dysfunction [24], chromosome abnormalities [25], DNA methylation and impaired epigenetic regulations [26, 27]. The developmental stage at which embryos are vitrified has an important effect on post-warming developmental capacity. Impaired blastocyst formation has been reported in mouse [28, 29], pig [30], rabbit [31] and cow [32] vitrified zygotes. An increased blastocyst development can be achieved when morulae are subjected to vitrification [28, 33]. However, zygote vitrification is more advantageous since it enables its use for alternative purposes such as genome editing. In the current study birth rate was not affected by vitrification. Even though this finding requires further investigation, it suggests a suitable outcome for the use of vitrified zygotes for CRISPR/Cas9 microinjection.

To evaluate the effect of vitrification in the mutation efficiency, heterodimer detection was used as a first approach in both blastocysts and pups which indicates that indels have been created as a consequence of the NHEJ repair system induced by Cas9 cleavage. As a second approach, Sanger sequencing was performed to confirm PAGE results in the live pups. Mutation rate results were very similar for both approaches, indel formation was greater in fresh microinjected zygotes, particularly in Experiment 2 where the difference was significant. It is important to mention that Sanger sequencing was more precise to detect 1bp indel. The rationale behind why vitrified zygotes were less prone to carry mutations than fresh ones deserve further investigation. Several reports have shown that DNA epigenetic changes, such as DNA methylation, genomic imprinting [34] and histone modification [35] are affected by vitrification. Therefore, we can speculate that vitrification could somehow affect the chromatin accessibility to CRISPR components, reducing its efficiency to produce the double strand break and/or the subsequent repair mechanisms. In future studies, the efficiency of ssODN or donor plasmid integration in fresh *vs*. vitrified-warmed zygote microinjection should be evaluated in the conditions used in this work. Moreover, the microinjection timing after warming of vitrified zygotes has to be taken into consideration, especially for knock in mice model generation [36]. The simplicity and high efficiency of CRISPR/Cas9 system to produce engineered mouse models may be improved even more by the optimization of the associated pipeline and accomplishment of 3R principles [37]. The possibility to maintain zygotes in a cryopreserved state, instead of having a great number of females and males to constantly produce zygotes, not only represent an operational advantage, but also reduce the necessity of rearing animals during the whole year in the facility. This kind of advantage should be taken into consideration in mice facilities producing genetically modified models, when the possibility of zygote cryobanking is available.

## Conclusion

Vitrified-warmed zygotes, using the minimum volume spatula method, are a suitable source of embryos for the generation of genetically modified mouse models through CRISPR/Cas9 microinjection. The possibility of zygote cryobanking to perform microinjection sessions according to the project requirements seems to be an interesting alternative to avoid the continuous breeding and maintenance of animals for zygote production. This novel strategy improves and simplifies the CRISPR pipeline enhancing the implementation of this technology.

## Supporting information

S1 Raw image Uncropped and unadjusted PAGE image. (PDF)

S1 Fig Representative sequencing alignment. (DOCX)

S1 Table Target gene information and sgRNA sequences. (DOCX)

S2 Table Target gene information and primer sequences. (DOCX)

S3 Table Experiment 2. Embryo survival, cleavage, pregnancy, birth and mutation rates of CRISPR/Cas9 microinjected fresh or vitrified/warmed C57BL/6J zygotes, separated by project. (DOCX)
