# Supplementary material for: Zygote cryobanking applied to CRISPR/Cas9 microinjection in mice
Source: PLoS One. 2024 Jul 9;19(7):e0306617. doi: 10.1371/journal.pone.0306617 (PMC11232997; doi:10.1371/journal.pone.0306617)
Supplement: S1 Raw image — (PDF) [file pone.0306617.s001.pdf]

S1_raw_image

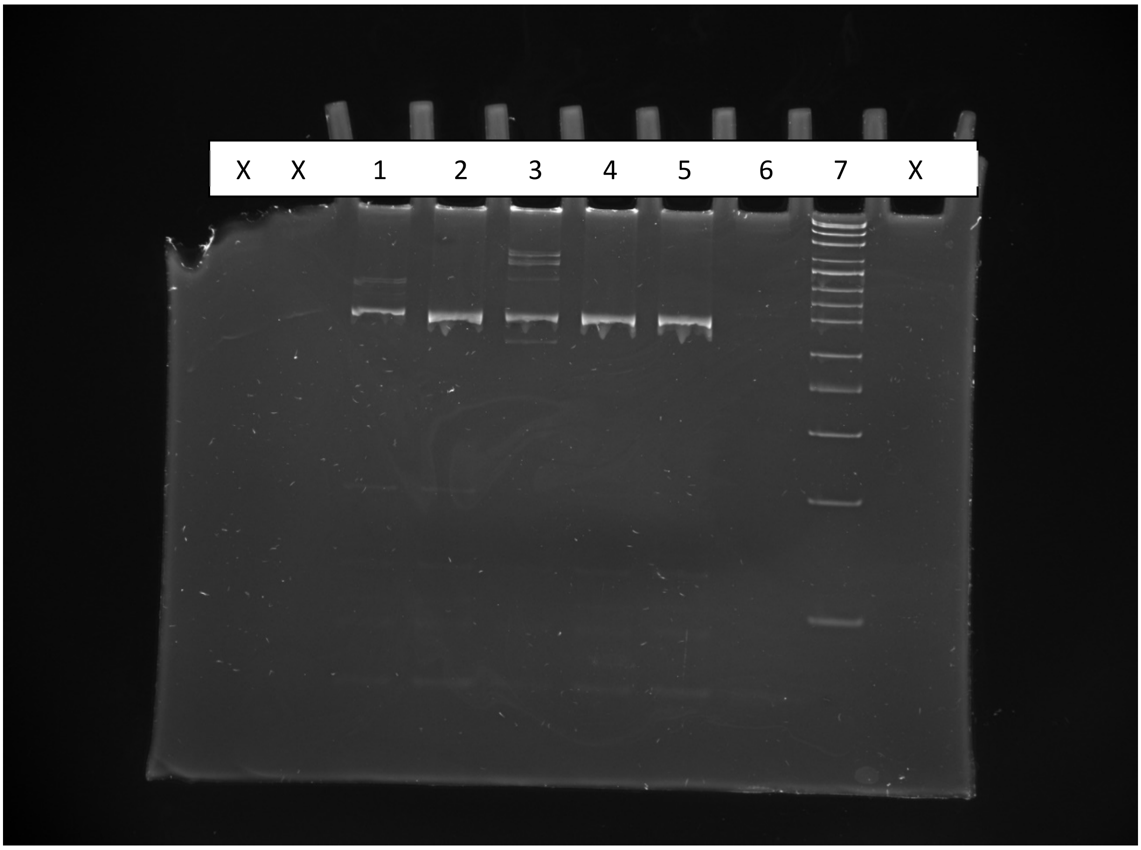

Uncropped and unadjusted image of Figure 3.

Loading order:

Line#1: sample 0

Line#2: sample 1

Line#3: sample 2

Line#4: sample 3

Line#5: wild-type sample

Line#6: PCR negative control

Line#7: 1 kb plus DNA ladder (Thermofisher)
