# Supplementary material for: Zygote cryobanking applied to CRISPR/Cas9 microinjection in mice
Source: PLoS One. 2024 Jul 9;19(7):e0306617. doi: 10.1371/journal.pone.0306617 (PMC11232997; doi:10.1371/journal.pone.0306617)
Supplement: S1 Fig — (DOCX) [file pone.0306617.s002.docx]

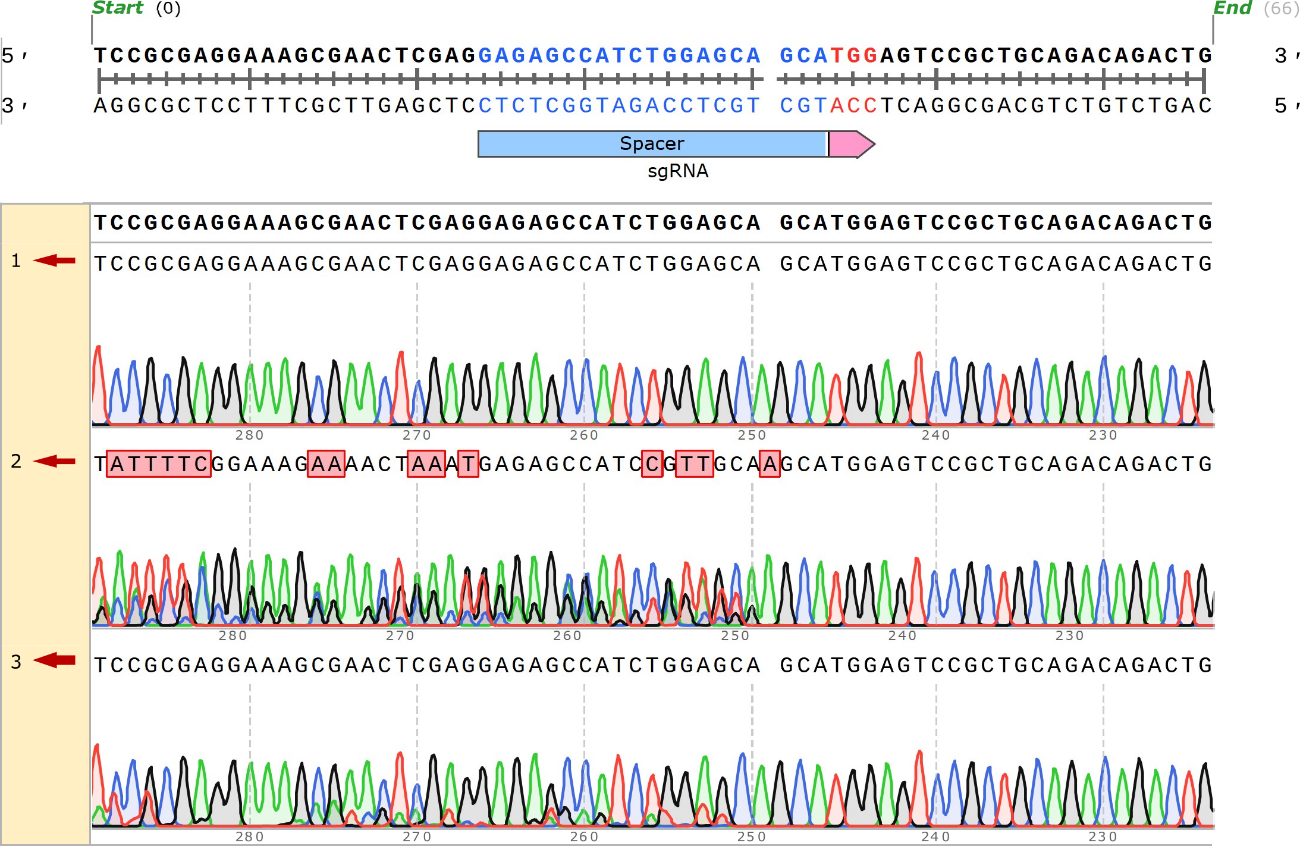


S1 Figure. The image shows an alignment of three sequences coming from different animals. Sample #1 has a WT genotype, while samples #2 and #3 have indels that caused multiple peaks in the chromatogram. These multiple peaks appear before the Cas9 cutting site (3bp prior to PAM sequence) because the samples were sequenced by a reverse primer, as symbolized by the red arrows pointing left next to the sample numbers.
