# Supplementary material for: Zygote cryobanking applied to CRISPR/Cas9 microinjection in mice
Source: PLoS One. 2024 Jul 9;19(7):e0306617. doi: 10.1371/journal.pone.0306617 (PMC11232997; doi:10.1371/journal.pone.0306617)
Supplement: S1 Table — (DOCX) [file pone.0306617.s003.docx]

| **Project #** | **Target gene** | **sgRNA name** | **sgRNA sequence** |
| --- | --- | --- | --- |
| 1 | *Syce1* | Syce1 sgRNA | UCUUUUUUGCUUUACCAAAGAGG |
| 2 | *Cd300lf* | Cd300lf sgRNA2 | GAGUUCUUAAGCCCCGACCUAGG |
| 3 | *Cdkn2a* | P16 sgRNA | GAGAGCCAUCUGGAGCAGCAUGG |
| 4 | *Cd300lf* | hCLM1 sgRNA1 | CAGCGGGAAGUGGCUAAAGGAGG |
|  | *Cd300lf* | hCLM1 sgRNA2 | GAUCACAGGUGAGCGAGCUCAGG |

**S1 Table. Target gene information and sgRNA sequences**

One sgRNA was used to target the corresponding gene in projects #1, 2, and 3, while two sgRNA were used in project #4.
