# Supplementary material for: Zygote cryobanking applied to CRISPR/Cas9 microinjection in mice
Source: PLoS One. 2024 Jul 9;19(7):e0306617. doi: 10.1371/journal.pone.0306617 (PMC11232997; doi:10.1371/journal.pone.0306617)
Supplement: S2 Table — (DOCX) [file pone.0306617.s004.docx]

**S2 Table. Target gene information and primer sequences**

| **Target gene** | **Primer name** | **Primer sequence** |
| --- | --- | --- |
|  |  | **PAGE analysis** |
| *Cd300lf* | 300F Fw | TTTGACACCCCTTCAGCTCC |
| *Cd300lf* | 300F Rv | CCAGGGCTCTCCTGTCTTTC |
| *Syce1* | Fw Syce1 | AGCTTCCTTTTTGCTTTGCAG |
| *Syce1* | Rv Syce1 | GGTTGGAGGAGAAGTCATGG |
| *Cdkn2a* | P16 Fw | CAGGTCAGGAGCAGAGTGTG |
| *Cdkn2a* | P16 Rv | GATGGGACACTCCTTGCCTA |
|  |  |  |
|  |  | **Sequencing analysis** |
| *Cd300lf* | 300F Fw * | TTTGACACCCCTTCAGCTCC |
| *Cd300lf* | 300F Rv * | CCAGGGCTCTCCTGTCTTTC |
| *Cdkn2a* | Fw ext 2 P16 | GGGGCTGTCCGATCCTTTAG |
| *Cdkn2a* | Rv ext 2 P16 * | GTTTAATGGGTGGCTCCGGT |

Primers identified with (*) are the sequencing primers.
