# Supplementary material for: Zygote cryobanking applied to CRISPR/Cas9 microinjection in mice
Source: PLoS One. 2024 Jul 9;19(7):e0306617. doi: 10.1371/journal.pone.0306617 (PMC11232997; doi:10.1371/journal.pone.0306617)
Supplement: S3 Table — (DOCX) [file pone.0306617.s005.docx]

| #Project; number of sessions | Group | No. of micro- injected zygotes | Embryo survival (live/micro- injected) | Cleavage rate (2-cell/live embryos) | Pregnancy rate (pregnant/ transferred females) | Birth rate  (live pups*/ transferred embryos) | Mutation rate^1^ (positive/ live pups**) | Mutation rate^2^ (positive/ live pups**) |
| --- | --- | --- | --- | --- | --- | --- | --- | --- |
| 2;  1 session | Fresh zygotes | 92 | 38.0%  (35/92) | 100.0%  (35/35) | 100.0%  (1/1) | 30.0%  (6/20) | 83.3%  (5/6) | 83.3%  (5/6) |
|  | Vitrified zygotes | 88 | 68.2%  (60/88) | 85.0%  (51/60) | 100.0%  (2/2) | 20.0%  (8/40) | 25.0%  (2/8) | 25.0%  (2/8) |
|  | *P-*value |  | <0.001 | 0.99 | 0.99 | 0.39 | 0.07 | 0.07 |
| 3;  4 sessions | Fresh zygotes | 389 | 53.5%  (208/389) | 68.3%  (142/208) | 62.5%  (5/8) | 8.8%  (12/136) | 80.0%  (8/10) | 90.0%  (9/10) |
|  | Vitrified zygotes | 383 | 61.4%  (235/383) | 62.6%  (147/235) | 50%  (4/8) | 8.7%  (12/138) | 33.3%  (4/12) | 50.0%  (6/12) |
|  | *P-*value |  | 0.27 | 0.21 | 0.62 | 0.97 | 0.05 | 0.08 |
| 4;  1 session | Fresh zygotes | 82 | 41.5%  (34/82) | 58.8%  (20/34) | 100.0%  (1/1) | 15.0%  (3/20) | 33.3%  (1/3) | 100.0%  (3/3) |
|  | Vitrified zygotes | 87 | 63.2%  (55/87) | 70.9%  (39/55) | 50.0%  (1/2) | 11.1%  (4/36) | 50.0%  (2/4) | 50.0%  (2/4) |
|  | *P-*value |  | 0.056 | 0.25 | 0.99 | 0.68 | 0.68 | 0.99 |

**S3 Table. Experiment 2. Embryo survival, cleavage, pregnancy, birth and mutation rates of CRISPR/Cas9 microinjected fresh or vitrified/warmed C57BL/6J zygotes, separated by project**.

Results are shown as percentages. Number of embryos are shown in parenthesis. *live pups 7 days after birth. ** live pups after 21 days after birth. Mutation rate^1^ refers to detection of DNA heterodimers in PAGE; mutation rate^2^ refers to detection of indels by Sanger sequencing.
